# Inflammatory biomarker score and cancer: A population-based prospective cohort study

**DOI:** 10.1186/s12885-016-2115-6

**Published:** 2016-02-10

**Authors:** Leavitt Morrison, Jari A. Laukkanen, Kimmo Ronkainen, Sudhir Kurl, Jussi Kauhanen, Adetunji T. Toriola

**Affiliations:** Department of Surgery, Division of Public Health Sciences, and Siteman Cancer Center, Washington University School of Medicine, St Louis, MO 63144 USA; Institute of Public Health and Clinical Nutrition, University of Eastern Finland, Kuopio, 70211 Finland; Department of Internal Medicine, Lapland Central Hospital, Rovaniemi, 96300 Finland

**Keywords:** Biomarkers, c-reactive protein, Inflammation, Leukocyte count, Cancer mortality

## Abstract

**Background:**

Inflammation is associated with cancer but there are conflicting reports on associations of biomarkers of inflammation with cancer risk and mortality. We investigated the associations of C-reactive protein (CRP) and leukocyte count with cancer risk and mortality using individual biomarkers, and an inflammatory score derived from both biomarkers.

**Methods:**

We conducted this analysis among 2,570 men enrolled in the population-based, prospective Kuopio Ischemic Heart Disease Risk Factor Study in Finland. During an average follow-up period of 26 years, 653 cancer cases and 287 cancer deaths occurred. We computed a z-score for each participant, with the combined z-score being the sum of each individual’s CRP and leukocyte z-scores. Multivariable-adjusted Cox proportional hazard model was used to evaluate associations with cancer risk and mortality.

**Results:**

Using individual biomarkers, elevated leukocyte count was associated with an increased risk of cancer (RR = 1.31, 95 % CI 1.04-1.66), and cancer mortality (RR=, 95 % CI 1.39, 0.98-1.97). The corresponding results for CRP were (RR = 1.23, 95 % CI 0.97-1.55) for risk and (RR = 1.15, 95 % CI 0.81-1.64) for cancer mortality. Associations of the biomarkers with cancer appeared to be more robust using the combined z-score. HRs comparing men within the highest z-score quartile to those within the lowest z-score quartiles were 1.47 (95 % CI 1.16-1.88, *p*-trend < 0.01) for cancer risk, and 1.48 (95 % CI 1.03-2.14, *p*-trend = 0.09) for cancer mortality.

**Conclusion:**

Our study suggests that inflammation is associated with cancer risk and mortality, and combining inflammatory biomarkers into a score is a robust method of elucidating this association.

## Background

Inflammation is associated with cancer risk and mortality [1, 2]. Several cancer types arise from local inflammatory states. For instance, inflammatory bowel disease [3], reflux esophagitis [4], and chronic pancreatitis [5] are associated with increased risk of colorectal, esophageal and pancreatic cancers, respectively. Nevertheless, there are conflicting reports on the associations of biomarkers of systemic inflammation with overall cancer risk and mortality. C-reactive protein and leukocyte counts are biomarkers of systemic inflammation that have been related to all-cause cancer and cancer mortality in previous studies [6–12]. While some studies have demonstrated positive associations between biomarkers of inflammation and overall cancer risk [6, 7] and mortality [8, 10, 12, 13], other studies have not reported any association [9, 14, 15]. These results demonstrate a need to further evaluate how biomarkers of systemic inflammation relate to cancer risk and mortality.

Because the biomarkers are systemic, associations between individual biomarkers and cancer may not be always apparent. One inflammatory marker may correctly classify some cases and misclassify others and this could contribute to the differences in results reported in previous studies. Hence, combining inflammatory biomarkers using an inflammatory score may provide a more accurate classification of inflammatory status [16]. Thus, an inflammatory score may be more robust compared to individual biomarkers in the assessment of cancer risk. To elucidate on the associations of inflammatory biomarkers with cancer, we investigated the associations of CRP and leukocyte count with cancer risk and mortality independently, as well as using a derived biomarker z-score combining both biomarkers. We hypothesize that combining CRP and leukocyte count would be a robust method of elucidating the associations of biomarkers with cancer risk and mortality.

## Methods

### Study population

This analysis was conducted among participants enrolled in a prospective cohort study from Eastern Finland; the Kuopio Ischemic Heart Disease Risk Factor Study (KIHD). Detailed description of this cohort has been provided in previous studies [17–19]. The KIHD was originally designed to investigate risk factors for cardiovascular diseases, and other health related outcomes in a population-based sample of middle-aged men from Eastern Finland. Baseline examinations were conducted between March 1984 and December 1989. The study group is a representative sample of men living in Kuopio and its surrounding rural communities who were aged 42, 48, 54 and 60 years at the time of baseline examination. Of the 3235 eligible men, 2682 (83 %) volunteered to participate and 198 men were excluded because of serious disease. For the present study, we further excluded men who had a history of cancer at baseline and those who had incomplete data. Hence, 2,570 men were available for the present analysis. Blood samples were collected from each participant once at baseline examination. Baseline blood samples were analysed for the C-reactive protein and leucocyte count.

### Cancer cases and cancer mortality

Incident cancer cases were derived from the Finnish Cancer Registry (FCR). The FCR is population-based, covers the whole of Finland and coverage is virtually complete with no loss to follow-up. For solid tumours; the registration is >99 % complete [20]. The FCR file containing personal identity codes is annually matched through computerized linkage with the cause of death register at the Statistics Finland so that dates and cause of deaths in cancer patients can be added to the FCR records [21]. The FCR file is also regularly linked with the Central Population Register to ensure that the personal identity codes are correct. Our study cohort was record linked with the FCR data using the 11-digit personal identity code mandatory to every resident of Finland since 1961. Outcomes were assessed annually with the help (linking) of the personal identity codes. All cancer diagnoses (*N* = 653) that occurred between the study entry (March 1984 to December 1989) and December 2012 were included. The most prevalent cancers were; prostate (*N* = 231), lung (*N* = 87) and colorectal (*N* = 83) cancers. Likewise, FCR file that contains personal identity codes is annually matched to the death register at the Statistics Finland using computerized linkages. This ensures that dates and causes of deaths are added to the FCR records [21]. It is also regularly linked with the Central Population Register to ensure that the personal identity codes are correct. Due to the complete follow up system of the Finnish population, there are no losses to follow-up [21]. During the follow-up period 287 cancer deaths were reported, mainly from prostate, lung, and colorectal cancers.

### Assessment of covariates

The number of cigarettes, cigars, and pipefuls of tobacco currently smoked daily, and duration of regular smoking in years were recorded with a self-administered questionnaire, which was checked by an interviewer [19, 22]. A subject was defined as a smoker if he had ever smoked on a regular basis and had smoked cigarettes, cigars, or pipe within the past 30 days. Life-long exposure to smoking (cigarette pack years) was estimated as the product of the number of smoking years and the number of tobacco products smoked daily until the time of baseline examination. Food consumption was assessed at the time of blood sampling during the baseline phase of the study. Subjects were instructed on the use of household measures for quantitative recording of their food intake during the 4 days of data collection [23]. A nutritionist gave the instructions and checked the completed food intake records. Dietary intake of foods and nutrients was calculated using NUTRICA software (version 2.5; National Public Health Institute, Turku). The software is compiled using mainly Finnish values of nutrient composition of foods, and takes into account losses of vitamins in food preparation. The nutrient composition of foods in the NUTRICA software used reflects data on vitamin contents of fruits and vegetables [23]. Body weight was measured using a balance scale and BMI was computed as the ratio of weight to the square of height (kg/m^2^). Cardiorespiratory fitness (maximal oxygen uptake, VO_2_max) was defined as the highest value or the plateau of directly measured oxygen consumption using a respiratory gas analyzer (Mijnhardt, the Netherlands and Medical Graphics, St. Paul, Minn.) [18]. One metabolic equivalent corresponds to oxygen uptake of 3.5 mL/kg/min. Alcohol consumption was assessed with a structured quantity-frequency method using the Nordic Alcohol Consumption Inventory [24]. The questionnaire assessed alcohol consumption over the preceding 12 months. Usual frequency of intake and usual dose per sitting (in glasses or bottles) were queried separately for each beverage (beer, wine, spirits) with a structured response form.

### Laboratory analysis

Laboratory assay for leukocyte count was undertaken at the Institute of Public Health, University of Kuopio. Blood leukocyte count was measured using a cell counter (Coulter Counter Electronics Model DN, Luton, UK). The between batch CV was below 4 %. CRP was quantified at the laboratory of Clinical Chemistry, Kuopio University Hospital. Serum C reactive protein concentration was measured with an immunometric assay (Immulite High Sensitivity C-reactive protein assay, DPC, Los Angeles, USA). The between batch coefficient of variation (CV) was 3.1 % at the CRP level of 3.2 mg/l. The between batch CV was below 5 %.

### Statistical analysis

We compared baseline characteristics by cancer status using Chi-square (Χ^2^) test for categorical variables and Wilcoxon rank sum for continuous variables. Continuous variables are reported as mean (standard deviation) while categorical variables are reported as frequencies (percentages).

Cox proportional hazard model was used to evaluate associations of the biomarkers with cancer risk and mortality. We categorized the biomarkers into quartiles based on the distribution in the study population. Relative Risks (RRs) and 95 % confidence intervals (95 % CI) were calculated for each biomarker. We evaluated the associations of the biomarkers with cancer risk and mortality in 3 models, adjusting for confounders. Confounders were selected if the p-value associated with the variable was <0.10 in the models. In model 1, we adjusted for only age at baseline examination. In model 2, we additionally adjusted for examination year, alcohol intake (grams/week), energy intake (KJ/4-day period) and cardiorespiratory fitness. In model 3, we adjusted for all the variables in model 2 as well as smoking (pack-years). Body mass index (BMI) was not included in the multivariable analysis, as the p-value for BMI in the model was >0.10 and including BMI did not have any impact on the effect estimates.

In addition to individual biomarker analysis, the biomarkers were analyzed together using a standardized z-score. To compute the z-score, each biomarker was normalized using a z-transformation. Z-score was computed for each participant based on the subject’s biomarker levels (x), study mean (μ) and study standard deviation (σ). We computed the z-scores thus; z-score = (x - μ)/σ. The combined z-score was the sum of the subject’s individual CRP and leukocyte z-scores. We categorized study participants into quartiles of the combined z-score and evaluated associations with cancer risk and mortality using a Cox proportional hazards model, adjusted for confounders.

All tests were two-sided with significance considered to be a *p*-value < 0.05. Tests for trend were assessed using the Wald test. Analyses were performed using SAS version 9.3 (SAS Institute, Cary, NC).

## Results

Table 1 describes baseline characteristics by incident cancer status. Six hundred and fifty three men developed cancer and 287 men died from cancer during an average follow-up period of 26 years (23–28). Men who developed cancer were more likely to be older (53.9 vs. 52.7 years, *p*-value < 0.01), smokers (37.7 % vs. 28.0 %, *p*-value < 0.01), consume more alcohol (81.2 vs. 73.7 g/week, *p*-value = 0.03), and have higher leukocyte counts (5.84 vs. 5.66 10^9^/L, *p*-value < 0.01). There was a modest correlation (*r* = 0.31, *p*-value < 0.01) between CRP and leukocyte count (data not shown).

**Table 1:** Baseline Characteristics of participants in the Kuopio Ischemic Heart Disease Risk. Factor Study (KIHD) in Eastern Finland by incident cancer status

Characteristics	No Cancer *N* =1,917	Cancer *N* = 653	*P*-value
Age, years	52.71(5.27)	53.91(4.53)	<0.01
Body mass index (kg/m^2^)			
<25	599 (31.25 %)	202 (30.93 %)	
25-29.99	984 (51.33 %)	345 (52.83 %)	
30-34.99	277 (14.45 %)	87 (13.32 %)	
≥35	57 (2.97 %)	19 (2.91 %)	
Smoking history			<0.01
Non-smoker	1380 (71.99 %)	407 (62.33 %)	
Smoker	537 (28.01 %)	246 (37.67 %)	
Pack-years			<0.01
<10 Pack-years	96 (5.01 %)	48 (7.35 %)	
10-20 Pack-years	121 (6.31 %)	43 (6.58 %)	
>20 Pack-years	320 (16.69 %)	155 (23.74 %)	
Alcohol consumption, g/week	73.68 (134.18)	81.20 (145.21)	0.03
Energy intake, KJ, (4 day mean)	9912.31 (2599.35)	9674.02 (2536.23)	0.03
Vegetable intake, g (4 day mean)	286.21 (126.58)	287.53 (122.45)	0.75
Fruit and berry intake, g (4 day mean)	161.30 (146.76)	157.69 (138.80)	0.81
Cardiorespiratory fitness (VO_2_ max)	30.61 (7.63)	30.06 (7.52)	0.05
Family history of any cancer			0.21
No	1465 (76.42 %)	483 (73.97 %)	
Yes	452 (23.58 %)	170 (26.03 %)	
Leukocyte count, 1,000/μl	5.66 (1.56)	5.84 (1.67)	<0.01
C-reactive protein, mg/l	2.43 (4.22)	2.44 (3.83)	0.23

Continuous values are presented as mean (standard deviation) and categorical values as number (%)

There were positive associations between CRP, leukocyte count, and cancer risk, which were attenuated after adjustment for smoking (Table 2). In age-adjusted analysis (model 1), men within the highest quartiles of CRP (RR = 1.49, 95 % CI 1.20-1.86, p-trend = 0.01) and leukocyte count (RR = 1.64, 95 % CI 1.32-2.03, p-trend = 0.01) had higher risks of cancer compared to those within the lowest quartiles. Similar results were observed in multivariable-adjusted analysis not adjusted for smoking (model 2). The HRs were 1.37 (95 % CI 1.09-1.72, p-trend = 0.05) for CRP, and 1.56 (95 % CI 1.25-1.94, p-trend = 0.02) for leukocyte count. However, the associations between CRP and leukocyte count and cancer risk were attenuated after further adjustment for smoking (model 3). RRs comparing men within the highest quartiles of CRP and leukocyte count to those within the lowest quartiles were 1.23 (95 % CI 0.97-1.55, p-trend = 0.18) and 1.31 (95 % CI 1.04-1.66, p-trend = 0.19), respectively.

**Table 2:** Hazard Ratios (HR) and 95 % Confidence Intervals (CI) of cancer risk by quartiles of biomarkers and z-score

	Q1	Q2	Q3	Q4	P-trend
Leukocyte Count					
No of Cases/Total	157/672	146/638	170/630	180/630	
Age-adjusted	1.0 (ref)	1.02 (0.82-1.28)	1.29 (1.03-1.60)	1.64 (1.32-2.03)	0.01
Multivariable^1^	1.0 (ref)	1.01 (0.80-1.26)	1.24 (0.99-1.54)	1.56 (1.25-1.94)	0.02
Multivariable^2^	1.0 (ref)	0.97 (0.77-1.22)	1.15 (0.92-1.43)	1.31 (1.04-1.66)	0.19
C-reactive protein					
No of Cases/Total	151/639	168/643	163/645	171/643	
Age-adjusted	1.0 (ref)	1.13 (0.90-1.40)	1.22 (0.97-1.52)	1.49 (1.20-1.86)	0.01
Multivariable^1^	1.0 (ref)	1.09 (0.88-1.37)	1.17 (0.93-1.46)	1.37 (1.09-1.72)	0.05
Multivariable^2^	1.0 (ref)	1.08 (0.87-1.35)	1.11 (0.88-1.39)	1.23 (0.97-1.55)	0.18
Z-score^3^					
No of Cases/Total	139/642	163/643	168/643	183/642	
Multivariable^2^	1.0 (ref)	1.18 (0.94-1.49)	1.26 (1.00-1.59)	1.47 (1.16-1.88)	<0.01

^1^Multivariable model adjusted for age, examination year, alcohol intake, cardiorespiratory fitness and energy intake

^2^Multivariable model adjusted for age, examination year, alcohol intake, cardiorespiratory fitness, energy intake and smoking

^3^Z-score was computed for each participant based on the subject’s biomarker levels (x), study mean (μ) and study standard deviation (σ)

We computed the individual z-scores thus; z-score = (x - μ)/σ. The combined z-score is the sum of the subject’s individual CRP and leukocyte z-scores

We observed similar associations with cancer mortality (Table 3). In the age-adjusted model, the HRs of cancer mortality comparing men within the fourth quartile of CRP and leukocyte count to those within the first quartile were 1.65 (95 % CI 1.18-2.31, p-trend = 0.02) and 2.09 (95 % CI 1.52-2.89, p-trend = 0.01), respectively. The HRs were 1.40 (95 % CI 0.99-1.99, p-trend = 0.43) for CRP and 1.88 (95 % CI 1.36-2.62, p-trend = 0.04) for leukocyte count in multivariable analyses not adjusted for smoking. As with cancer risk, the further adjustments for smoking attenuated the observed associations between biomarkers and cancer mortality; RR = 1.15 (95 % CI 0.81-1.64, p-trend = 0.43) for CRP, and RR = 1.39 (95 % CI 0.98-1.97, p-trend = 0.42) for leukocyte count.

**Table 3:** Hazard Ratios (HR) and 95 % Confidence Intervals (CI) of cancer mortality by quartiles of biomarkers and z-score

	Q1	Q2	Q3	Q4	P-trend
Leukocyte Count					
No of Cases/Total	62/672	54/638	79/630	92/630	
Age-adjusted	1.0 (ref)	0.95 (0.66-1.37)	1.48 (1.06-2.07)	2.09 (1.52-2.89)	0.01
Multivariable^a^	1.0 (ref)	0.93 (0.65-1.34)	1.38 (0.99-1.93)	1.88 (1.36-2.62)	0.04
Multivariable^b^	1.0 (ref)	0.87 (0.60-1.25)	1.20 (0.85-1.68)	1.39 (0.98-1.97)	0.42
C-reactive protein					
No of Cases/Total	61/639	73/643	75/645	78/643	
Age-adjusted	1.0 (ref)	1.18 (0.84-1.66)	1.37 (0.97-1.92)	1.65 (1.18-2.31)	0.02
Multivariable^a^	1.0 (ref)	1.13 (0.80-1.59)	1.25 (0.88-1.75)	1.40 (0.99-1.99)	0.12
Multivariable^b^	1.0 (ref)	1.10 (0.78-1.55)	1.12 (0.79-1.59)	1.15 (0.81-1.64)	0.43
Z-score					
No of Cases/Total	54/642	65/643	77/643	91/642	
Multivariable^b^	1.0 (ref)	1.12 (0.78-1.61)	1.30 (0.91-1.86)	1.48 (1.03-2.14)	0.09

^a^Multivariable model adjusted for age, examination year, alcohol intake, cardiorespiratory fitness and energy intake

^b^Multivariable model adjusted for age, examination year, alcohol intake, cardiorespiratory fitness, energy intake and smoking

In multivariable analysis conducted using z-score, we observed consistent positive associations with cancer risk and mortality in all models, regardless of adjustment for smoking. In multivariable model adjusted for all confounding factors, men within the highest z-score quartile had a higher risk of cancer when compared to those within the lowest z-score quartile; RR = 1.47 (95 % CI 1.16-1.88, p-trend < 0.01). Similarly, men within the highest z-score quartile had increased cancer mortality; RR of 1.48 (95 % CI 1.03-2.14), although the trend test was not statistically significant (p-trend = 0.09). We observed no evidence of interaction by smoking status for cancer risk and mortality for analyses using the individual biomarkers and the z-score. For instance, the multivariable adjusted HR for cancer incidence among men within the fourth quartile of z-score compared to those within the first quartile was 1.32 (95 % CI 0.96-1.80) for non-smokers and 1.46 (95 % CI 0.93-2.29) for smokers (data not shown). In addition, there was no interaction between CRP and leucocyte count.

## Discussion

In this population-based prospective cohort study with long follow-up, a combined inflammatory score was associated with increased risk of incident cancer. Data from this study further support the growing evidence that inflammation plays a role in cancer risk and mortality. Furthermore, our results indicate that combining biomarkers of inflammation is robust method of elucidating the associations of inflammation with cancer risk and mortality.

Similar to our findings using individual biomarkers, a recent analysis within the European Prospective Investigation of Cancer (EPIC)-Norfolk cohort demonstrated an association between CRP and cancer mortality although it was not significant when other confounders were taken in account [9]. In sex-stratified analysis [9], men within the highest CRP levels had a non-statistically significant 1.17-fold increased risk of cancer death compared to men with low levels of CRP, similar to what we observed in our study (RR = 1.23). In another study among males in 3 large cohorts from the United Kingdom, CRP and leukocyte counts were associated with increased risk of smoking-related cancers but the associations were confounded by smoking such that adjustment for smoking completely attenuated the associations [15]. Smoking is associated with changes in CRP concentrations and is thus an important confounder when relating CRP to cancer risk or mortality [25]. Likewise, a study among Koreans did not report any associations between leukocyte count and cancer death among men and women, although elevated leukocyte count was associated all-cause mortality [14]. Hence, our observations of positive associations in analyses using z-scores underscore the utility of combining inflammatory biomarkers into a score when evaluating the associations of inflammatory biomarkers with cancer.

Other studies within the Swedish Apolipoprotein MOrtality RISk (AMORIS) and The National Health and Nutrition Examination Survey (NHANES) III have also evaluated the utility of biomarker scores in relation to overall cancer risk and mortality [26–28]. However, our methods of deriving the biomarker scores differed compared with the AMORIS and NHANES III study. In one of the studies conducted within the AMORIS cohort, the authors derived an inflammation-based predictive score (IPS) using CRP and leukocyte count, where they classified the cohort into 2 groups and assigned a score of 1 if an individual had both CRP and leukocyte count above a clinical cut-off point and a score of 0 otherwise [26]. Having an IPS score of 1 was associated with increased overall cancer risk compared with a score of 0. The authors, however, did not adjust for smoking, which is an important confounder and did not evaluate cancer mortality associations. In the NHANES III study and another study within the AMORIS cohort, a quantitative score, ranging from 0-4, derived by adding a number of biomarkers (CRP, albumin, gamma-glutamyl transferase, and HDL cholesterol) with abnormal values was positively associated with cancer mortality [27, 28]. These biomarkers, however, have different mechanistic importance, with CRP considered the sole biomarker of systemic inflammation of the 4 biomarkers used. Although there are key differences compared with our current study in how the scores were derived, these offer robust evidence for the utility of a biomarker score when evaluating cancer risk and mortality.

On the other hand, some studies have reported positive associations of CRP and leukocyte count with cancer risk and mortality using the individual markers alone and the reasons may be due to differences in age and gender compositions the study populations [10, 12]. Our study is limited to men while these studies had men and women in their cohorts. In addition, participants in the Health Aging and Body Composition cohort are much older compared with those in our study cohort with a minimum age at recruitment of 70 years, compared with 48 years in our study.

Our study has the following limitations. We related CRP concentration and leukocyte count at one-time point to cancer risk and mortality. This might underestimate any associations, especially between CRP and cancer. Published data indicate that relating a single measurement of CRP to cancer risk underestimates the true strength of the association [29]. Our study cohort consisted of only Caucasian White men; hence our results may not be generalizable to women and other racial groups. We evaluated the associations of the biomarkers with overall cancer risk and mortality and some of the cancer sites included may not have an inflammatory origin. This may attenuate any possible associations. Nevertheless, three cancers; prostate (*N* = 231), lung (*N* = 87) and colorectal (*N* = 83) constitute 61 % of the incident cancer cases in our cohort. Inflammation has been shown to contribute to these three cancers and it is possible that these 3 cancers drive the overall associations we observed in our study.

In spite of the limitations, our study has many strengths. It is population-based, prospective in nature, with a long follow-up period and virtually no loss-to follow up. Additionally, the Finnish Cancer Registry is >99 % complete and is annually linked to the national cause of death registry. Because of this, we were able to include all deaths that occurred in the cohort in our analysis. Furthermore, many confounders were measured in the KIHD study, which allowed us to comprehensively explore their roles in the observed associations.

## Conclusions

In summary, our data support evidence linking inflammation with cancer and suggest that combining inflammatory biomarkers into a score is a robust method of elucidating the associations of inflammatory biomarkers with cancer risk and mortality. These results require confirmation. In addition, the utility of including additional inflammatory biomarkers to derive a clinically useful risk score could be explored in future prospective studies.

## Ethics approval and consent to participate

The Research Ethics Committee of the University of Kuopio approved the study protocol. Each participant gave a written informed consent. Access to the database was approved by the Research Ethics Committee at the University of Eastern Finland, Kuopio, Finland.

## Abbreviations

AMORIS: Apolipoprotein MOrtality RISk
BMI: body mass index
CV: coefficient of variation
CI: confidence intervals
CRP: C-reactive protein
EPIC: European Prospective Investigation of Cancer
FCR: Finnish Cancer Registry
KIHD: Kuopio Ischemic Heart Disease Risk Factor Study
NHANES: National Health and Nutrition Examination Survey
RR: Relative Risk
